# Lipidomics analysis of phospholipid profiles and oxidative stability in pan-fried beef patties incorporating sacha inchi leaf extracts

**DOI:** 10.1038/s41598-025-13267-x

**Published:** 2025-08-02

**Authors:** Putri Widyanti Harlina, Asad Nawaz, Siti Afina Sabrina, Ernisa Adha Nur’isma, Fang Geng, Raheel Shahzad, Mohamad Rafi, Vevi Maritha, Bara Yudhistira, Ahmad Ni’matullah Al-Baarri, Heni Radiani Arifin, Edy Subroto, Elazmanawati Lembong, Vira P. Yarlina

**Affiliations:** 1 Department of Food Industrial Technology, Faculty of Agro-Industrial Technology, Universitas Padjadjaran, Bandung, 45363 Indonesia; 2 Hunan Engineering Technology Research Center for Comprehensive Development and Utilization of Biomass Resources, College of Chemistry and Bioengineering, Hunan University of Science and Engineering, Yongzhou, 425199 China; 3 Master Program of Agro-Industrial Technology, Faculty of Agro-Industrial Technology, Universitas Padjadjaran, Bandung, Indonesia; 4 Meat Processing Key Laboratory of Sichuan Province, School of Food and Biological Engineering, Chengdu University, Chengdu, 610106 China; 5 Research Center for Genetic Engineering, National Research and Innovation Agency (BRIN), KST-Cibinong, JI Raya Bogor KM 46, Cibinong, 16911 Indonesia; 6 Department of Chemistry, Faculty of Mathematics and Natural Sciences, IPB University, Jalan Tanjung Kampus IPB Dramaga, Bogor, 16680 Indonesia; 7 Pharmacy Study Program, Faculty of Health and Science, Universitas PGRI Madiun, Madiun, Indonesia; 8 Department of Food Science and Technology, Sebelas Maret University, Surakarta City, Central Java Indonesia; 9 Department of Food Technology, Faculty of Animal and Agricultural Sciences, Universitas Diponegoro, Semarang, Indonesia

**Keywords:** *Plukenetia volubilis*, Lipid oxidation, Antioxidant, Lipidomics, Frying, Lipidomics, Lipids, Lipidomics, Lipids

## Abstract

High-temperature cooking methods, such as pan-frying, often accelerate lipid oxidation in meat products, negatively affecting their nutritional quality and safety; thus, natural antioxidants, such as those found in sacha inchi (*Plukenetia volubilis*) leaves, could be beneficial in mitigating this issue. Beef patties were additioned with sacha inchi leaf extracts at concentrations of 0.5%, 1.0%, and 1.5%, focusing on their antioxidant effects, lipid oxidation, and lipid profiles. The results showed that the addition of sacha inchi leaf extracts to pan-fried beef patties significantly reduced lipid oxidation. Higher concentrations of extracts correlated with increased antioxidant activity. Lipid profiling had distinct differences between samples, with the 1.5% of sacha inchi leaf extracts concentration showing the most promising results. Specifically, this concentration was characterized by elevated levels of LPC (*Lysophosphatidylcholine*)(18:2), LPE(*Lysophosphatidylethanolamine*)(18:2), and PA (*Phosphatidic Acid*)(27:2/8:0). These findings suggest that the sacha inchi leaf extracts not only mitigate lipid oxidation but also enhance the nutritional value of beef patties by influencing their lipid composition.

**Supplementary Information:**

The online version contains supplementary material available at 10.1038/s41598-025-13267-x.

## Introduction

Globally, there is a significant change in consumer consumption patterns, activities, and nutritional status. The increasing pace of modern life, characterized by high mobility and demanding work schedules, has made people have less time for eating. Consequently, dietary habits have become more westernized with a lower consumption of fiber and a higher intake of fats. This has led to an increase in demand for fast food such as meat products, which are obtained by subjecting raw meat to various processing methods and transforming into consumable food items or other forms. The processing methods often include heat treatments such as boiling, frying, baking, steaming, and grilling to extend shelf life and enhance the sensory qualities of products^1^. Meat and processed products such as beef patties are widely consumed due to their nutritional content, including essential vitamins, fatty acids, amino acids, and minerals^2^.

Beef patties can be categorized as fast food products made from various animal species such as beef, pork, and chicken that can be processed with different methods and specific organoleptic characteristics^3^. These products are added with some food ingredients to enhance taste, aroma, color, and appearance^4^The pan-frying method of beef patties includes specific temperature conditions that can induce substantial lipid changes due to oxidation and explored the association between lipid oxidation and cooking temperature in processed meat products where a directly proportional relationship was found^5–7^. The high oxidative processes were correlated with adverse health outcomes such as cardiovascular disease^8^.

High cooking temperatures is related to lipid oxidation in processed meat products, affecting both sensory attributes and nutritional content. Macho-González et al.^9^ and Wang et al.^10^ reported that lipid oxidation increased at elevated temperatures potentially contributing to disease hypertension, obesity, and cancer. It was also reported that the efficacy of various antioxidants from black, green, and pink pepper^11^black rice water extracts^12^and mulberry leaf^13^ could reduce lipid oxidation in processed meat products. Antioxidants help preserve the nutritional value and sensory attributes^14^preventing degenerative diseases related to free radicals, such as cardiovascular, stroke, and cancer^15^. Although synthetic antioxidants are commonly added to meat, their use has become less accepted, leading to an increased interest in natural antioxidants from plants, such as leaf. Among these plants, sacha inchi leaf (*Plukenetia volubilis*) contains terpenoids, saponins, phenolics, and other substances with antioxidant properties^16^.

The lipidomics method can be used to determine the quality of processed meat products by evaluating their lipid profile. Although the method has been used widely for various application, there is still limited information on lipidomics profile in meat products^17^. Lipidomics analysis can be carried out using specific instrument capable of identifying metabolites and lipids such as Ultra-High-Performance Liquid Chromatography-High Resolution Mass Spectrometry (UHPLC-HRMS). The instrument is suitable due to sensitivity in analyzing lipids and the ability to provide a comprehensive lipid profile, simple sample preparation, and accurate identification^18^.

Despite the abundant potential, there is no information on using sacha inchi leaf in meat to improve the quality of processed products. Therefore, this study aimed to explore the effects of adding sacha inchi leaf extracts on lipid profile and oxidation in beef patties.

## Materials and methods

Beef chucks, starch and dried sacha inchi leaf (*Plukenetia volubilis*) were obtained from a local market in Bandung, Indonesia. The other reagents were DPPH (1,1-diphenyl-2-picrylhydrazyl), HCl (Hydrochloric acid), potassium iodide, acetic acid, ethanol, chloroform, Na_2_S_2_O_3_, methanol, anhydrous sodium sulfate, TBA (Thiobarbituric Acid), formic acid, and acetonitrile LC-MS grade, which were all Pro Analysis quality chemical reagents (Merck, Germany).

### Methods

#### Extraction of sacha inchi leaf

Samples of dried sacha inchi leaf were ground into a fine powder before extraction. A maceration method was applied using a sample-to-ethanol 96% ratio of 1:10 (w/v). The mixture was macerated at room temperature in the dark for 3 days. Subsequently, the macerate was filtered using Whatman filter paper No. 1^19^. The filtrate was evaporated at 50° C and 80 rpm, while extracts were stored in a chiller at 4–5° C^19^.

#### Beef patties burger preparation

Beef was ground using a meat grinder and divided into four groups, namely control, and sacha inchi extracts at varying concentrations of 0.5%, 1.0%, and 1.5%. The concentrations of sacha inchi leaf extracts (0.5%, 1.0%, and 1.5%) were selected based on preliminary sensory evaluation. Higher concentrations (≥ 2.0%) introduced undesirable sensory attributes, particularly bitterness and herbal aftertaste, affecting consumer acceptability. Subsequently, beef patties weighing 30.00 ± 0.10 g were formed and cooking was conducted in a non-stick pan on a stovetop at 180° C for 4 min per side using 30 mL of cooking oil to prevent sticking. The cooked patties were stored at -18° C until further analysis^20^.

#### Determination of antioxidant activity

According to Gulcin & Alwasel^21^a spectrophotometric method, DPPH was used to determine antioxidant activity. Extraction was carried out by dissolving beef patties sample with 95% ethanol in a ratio of 1:10 (w/v) and extracting at 60° C in a water bath with continuous shaking at a speed of 170 r/min for 2 h. The obtained slurry was centrifuged at 10.000 × g for 10 min (Model 3740, Kubota, Osaka, Japan). The supernatant was used for antioxidant activity analysis and antioxidant activity was evaluated by free radical scavenging activity (DPPH method) to produce a 0.002% concentration of DPPH. Each test tube was filled with varying quantities of supernatant and the total volume was increased to 2 mL. After adding 2 mL of DPPH solution to each test tube, the solutions were left in the dark for half an hour, and every sample was examined three times. A UV-Visible spectrophotometer (NanoDrop 2000 C spectrophotometer, Thermo Fisher Scientific, USA) was used to measure optical density at 517 nm, while DPPH and ethanol were combined as control. The method followed the procedures of Harlina et al.^22^ with slight modification using the formula

1 $$\:\text{\%}\:\text{I}\text{n}\text{h}\text{i}\text{b}\text{i}\text{t}\text{i}\text{o}\text{n}\:\text{o}\text{f}\:\text{D}\text{P}\text{P}\text{H}\:\text{a}\text{c}\text{t}\text{i}\text{v}\text{i}\text{t}\text{y}\:=\frac{\text{A}-\text{B}}{\text{A}}\times 100$$

where: A = Optical density of control; B = Optical density of sample.

In this formula, A control refers to the absorbance of DPPH solution with ethanol whereas A sample refers to the absorbance of DPPH solution absorbed in the sample. In this manner, a calculation of the percentage of DPPH radical scavenging activity for each sample was done along with IC_50_ (mg/L). The lower the IC_50_ value of the samples, the greater the antioxidant activity.

#### Peroxide value in beef patties

The test was carried out using the iodometric titration method^23^. A total of 5 g of sample was weighed and heated in a 250 mL Erlenmeyer flask with a water bath at 60° C for 3 min for fat melting, followed by stirring for 3 min with 30 mL of acetic acid: chloroform solution (3:2 v/v) to dissolve the fat. The sample obtained was filtered using Whatman filter paper under vacuum added with 0.5 mL of saturated potassium iodide solution and shaken for 1 min, followed by 30 mL of distilled water and 0.5 mL of 1% starch indicator. Titration was performed with 0.1 N Na_2_S_2_O_3_ until the initial color of the sample disappeared or turned white. The calculation of peroxide value is as follows:

2 $$\:PV\:\left(\frac{mEq\text{O}2}{kg}\right)=\:\frac{S\:\times\:N}{W}\times\:1000$$

Information: S = titration volume (mL); N = normality of Na_2_S_2_O_3_; W = sample weight (g); Peroxide Value (PV): mEq O₂/kg.

#### TBA analysis on beef patties

The TBA test was carried out using the distillation method^24^. A total of 10 g of sample was weighed and put into a sharpened Kjeldahl flask. Next, 50 mL of distilled water was added to the sample, rinse using 47.5 mL of distilled water, and 2.5 mL of 4 M HCl was added to adjust a pH of 1.5. Distillation was carried out until 50 mL of distillate was collected in the Erlenmeyer flask. 5 mL of the collected distillate was pipetted into a test tube and 5 mL of 0.02 N TBA was added. Then, the sample was homogenized and heated in a water bath at 90° C for 35 min until it turned reddish. The absorbance of the sample was read with a spectrophotometer at a wavelength of 528 nm. The TBA number is calculated using the following formula.

3 $$\:\text{N}\text{u}\text{m}\text{b}\text{e}\text{r}\:\text{T}\text{B}\text{A}=\text{7,8}\:\times\:A$$

Information: 7.8 = TBA MDA number/kg sample; A = absorbance. TBA value: mg MDA/kg.

#### Extraction of lipids from meat

Lipid extraction referred to the method used by Harlina et al.^25^where 50 g of material was homogenized in a mixture of chloroform: methanol: distilled water (120:120:60 v/v/v) at a speed of 11,000 g for 2 min using a homogenizer. This combination was ultrasonified for 30 min at 20 °C with 80% capacity. A Buchner filter funnel was used to filter the mixture. In an Erlenmeyer flask, 2 g of anhydrous sodium sulfate was added to the chloroform phase (bottom layer) to remove water and salt residue. Whatman No. 1 paper was used to decant and filter lipid components in chloroform. The evaporator was initially washed with nitrogen prior to starting the rotary. Before analysis, the lipids were kept in vials at -20° C in nitrogen.

### Lipidomics analysis

Lipidomics analysis was carried out using UHPLC-HRMS in the Advance Research Laboratory IPB University. Before injecting the sample into the instrument, 5 mg of the lipid extracts sample was dissolved in 1 ml of methanol and filtered with a 0.2 μm Nylon membrane. UHPLC conditions include C18 Accucore (Thermo Scientific) 100 × 2.1 mm x 1.5 μm stationary phase. Binary solvent was used as a mobile phase, divided into A and B. Specifically, phase A was acetonitrile: water (60:40), 10 mol/L ammonium acetate, and phase B comprised isopropanol: acetonitrile (90:10), 10 mol/L ammonium acetate. A 2 µL of the sample was injected with a flow rate of 0.2 µL/minute for 35 min, with a column temperature of 30 °C and sample at 10 °C. The data was read at the mass interval m/z 100–1500 m/z and spectra fragments were obtained at resolutions of 70,000 and 17,500, voltage 3000 volts, capillary temperature 320 °C, heat temperature 300 °C, additional gas flow 3 Arb, and sheath gas flow 15 Arb. Spectra data processing was performed directly by matching LipidSeacrh library data. The detailed parameters of the LC gradient and MS instrument are shown in Supplementary Table S1, as described by Maritha et al.^18^ with slight modifications. Lipid identification and analysis were performed using LipidSearch 4.1.16 (Thermo Fisher, CA). The UHPLC-HRMS data obtained were followed by matching using the LipidSearch database to detect lipid compounds and structures.

### Determination texture and color analysis

Texture attributes (hardness, adhesiveness, springiness, cohesiveness, gumminess, chewiness, resilience) were measured using a Texture Analyzer (TA.XT2i, Stable Micro Systems, UK) based on the Texture Profile Analysis (TPA) method^26^. Color values (L*, a*, b*) were measured using a colorimeter (CR-400, Konica Minolta, Japan)^27^. All measurements were performed in triplicate.

### Statistical and chemometric analysis

Data were analyzed using IBM SPSS Statistics 25.0 software. One-way ANOVA was used to determine significant differences among groups, followed by Duncan’s multiple range tests at a 95% confidence level. Chemometric analyses, including Principal Component Analysis (PCA) and Partial Least Squares-Discriminant Analysis (PLS-DA), were performed using MetaboAnalyst 6.0 software (https://www.metaboanalyst.ca/).

## Results and discussion

### Effects of different concentrations of Sacha Inchi leaf extracts on lipid oxidation in beef patties

The study performed evaluation of lipid oxidation by determining both primary and secondary oxidation products in beef patties. This primary oxidation included peroxides value were evaluated using iodometric titration and the TBA test for secondary oxidation products^28^.

**Fig. 1:**
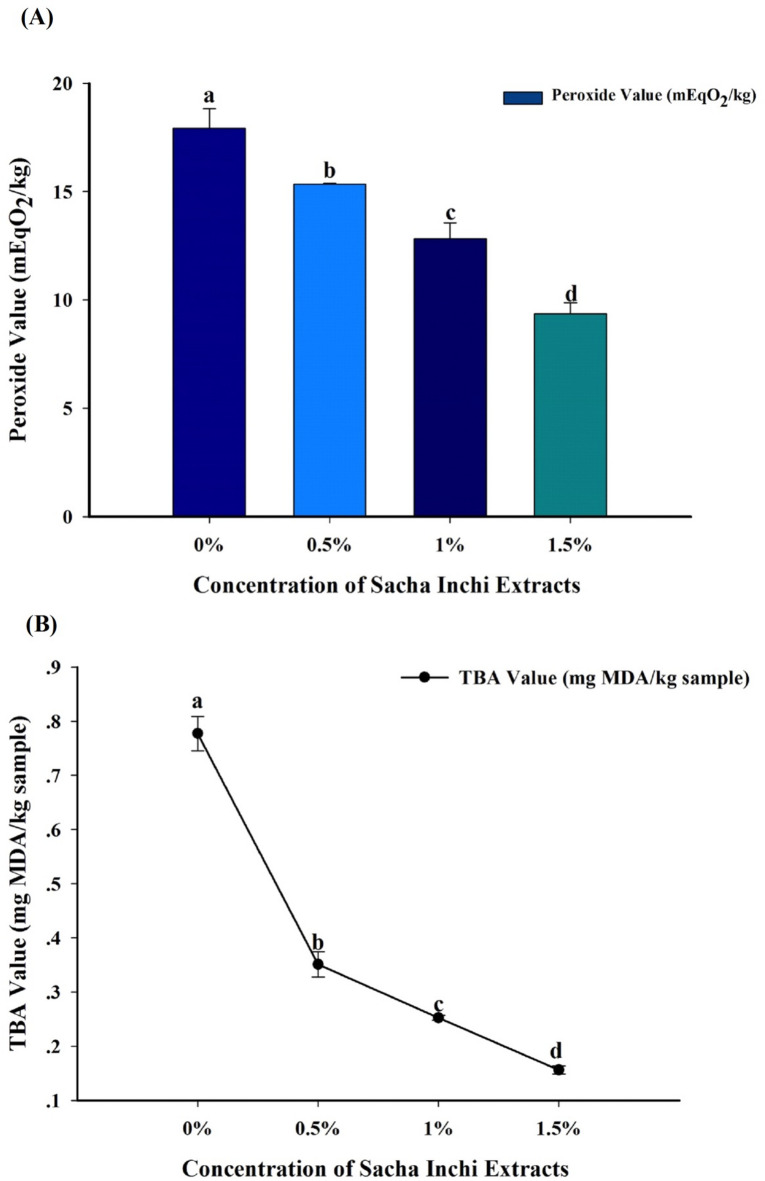
(**A**) Peroxide value and (**B**) TBA value in beef patties supplemented with different concentrations of sacha inchi leaf extracts. Values are presented as means ± standard deviations (*n* = 3). Bars and lines marked by different letters (a, b, c, d) indicate significant differences (*p* ≤ 0.05) as determined by one-way ANOVA followed by Duncan’s multiple range test.

The PV results showed significant variation in lipid oxidation according to distinctive quantities of sacha inchi leaf extracts. It was observed that when the extracts were added, peroxide value of samples significantly decreased (*p* < 0.05). The control sample showed the highest peroxide value followed by varying concentrations of 0.5%, 1.0%, and 1.5% extracts with values ranging from 17.92 to 9.37 mEqO_2_/kg, as presented in Fig. 1. According to Rahman et al.^29^the permissible peroxide value for beef and its derivatives is ≤ 25 mEqO_2_/kg. All samples in this investigation were within the permitted range, with the maximum value of 17.92 mEqO_2_/kg identified in the control sample.

The measurement of peroxide value alone is inadequate for a thorough assessment of lipid oxidation, as it solely quantifies primary oxidation products. Hydroperoxides, the principal oxidation products, could easily breakdown into secondary compounds. Therefore, secondary oxidation products, including MDA, were assessed using the thiobarbituric acid (TBA) test. TBA values decreased significantly (*p* ≤ 0.05) with increasing concentrations of sacha inchi leaf extracts, supporting the peroxide values. The highest TBA value was observed in the control sample, followed by samples with 0.5%, 1.0%, and 1.5% extracts (0.78 − 0.15 mg MDA/kg), as shown in Fig. 1. Zhang et al.^30^ reported that people typically tolerate beef patties with MDA levels ranging from 2 to 3 mg/kg. The European Food Safety Authority Scientific Committee has established a toxicological threshold of 30 mg MDA per kg of body weight per day^31^. MDA, generated in oils and fried foods, can be absorbed through the digestive system into the bloodstream, presenting possible health hazards^32^. At higher concentrations, the observed decrease in lipid oxidation was attributed to antioxidant properties, which contributed hydrogen atoms to neutralize free radicals, disrupting the lipid oxidation chain reaction^10^.

Lipid oxidation is a well-recognized concern in thermally processed meat products due to its negative impact on quality, nutrition, and health implications. Several studies have highlighted the potential of plant-based antioxidants to mitigate this oxidative stress. For example, Martini et al.^11^ demonstrated significant reductions in lipid oxidation markers when using pepper extracts during meat cooking, emphasizing the effectiveness of phenolic-rich plant extracts. Likewise, Prommachart et al.^12^ reported improved oxidative stability and sensory quality in beef patties treated with black rice extracts, corroborating our findings that natural antioxidants effectively counteract oxidative processes in meat systems. Furthermore, Xu et al.^13^ identified mulberry leaf extracts as effective inhibitors of lipid oxidation and heterocyclic amine formation during pan-frying, reinforcing the relevance of our findings with sacha inchi leaf extracts, another unconventional plant-derived antioxidant source.

### Effects of different concentrations of Sacha Inchi leaf extracts on antioxidant activity in beef patties

Beef patties with the sacha inchi leaf extracts were subjected to DPPH assay to evaluate their antioxidant potential. A color change from purple to yellow or colorless was observed after 30 min incubation in the samples reacted with DPPH. The results indicated a significant increase (*p* ≤ 0.05) in antioxidant activity of beef patties following the addition of sacha inchi leaf extracts, as demonstrated by the decrease in IC_50_ values shown in Table 1. Lower IC_50_ values indicate stronger antioxidant activity. The highest IC_50_ values were observed in samples with 0.5% sacha inchi leaf extracts. This was followed by 1.0% and 1.5%, with values ranging from 36494.76 to 16545.37 mg/L. In this study, the IC_50_ value of sacha inchi leaf extracts was 350.73 mg/L. According to Wuttisin et al.^33^the IC_50_ values of sacha inchi leaf extracts determined by the DPPH method ranged from 134.35 to 149.42 mg/L, showing moderate antioxidant activity. The presence of antioxidants in beef patties could contribute to preventing lipid oxidation. Antioxidants effectively scavenge free radicals formed in food by donating a hydrogen atom. This process stabilized the free radicals, thereby reducing oxidative damage^34^.

**Table 1:** IC_50_ value of beef patties samples.

Samples	Concentration (mg/L)	IC_50_ (mg/L)
Pure Sacha Inchi Extract	950	350,73 ± 1,210^a^
Beef Patty + SI 0,5%	95,000	36494,76 ± 82,408^b^
Beef Patty + SI 1,0%	60,000	27648,07 ± 22,106^c^
Beef Patty + SI 1,5%	45,000	16545,37 ± 67,792^d^

Values are presented as mean ± standard deviation and were analyzed using one-way ANOVA (*p* ≤ 0.05).

Different letters within the same column indicate significant differences according to Duncan’s multiple range tests at the 95% confidence level.

Pure Sacha Inchi Extract refers to the extract alone, used as a reference to evaluate its inherent antioxidant potency in the DPPH assay. Lower IC_50_ values indicate stronger antioxidant activity.

SI = Sacha Inchi.

Although high-temperature cooking at 180 °C reduces the antioxidant activity due to thermal degradation of phenolic and flavonoid compounds^35^the addition of sacha inchi leaf extracts still provides significant protective effects against lipid oxidation. Despite partial heat-induced degradation, sufficient bioactive compounds remain active after cooking, effectively lowering lipid oxidation parameters such as POV and TBA values (as shown in Fig. 1). Similar observations were also reported by Hihat et al.^36^ who noted that bioactive compounds could partially degrade but still retain antioxidant properties sufficient to inhibit oxidative deterioration during high-temperature processing.

### Correlation among lipid oxidation, antioxidant activity, and quality parameters in beef patties supplemented with sacha inchi leaf extracts

**Fig. 2:**
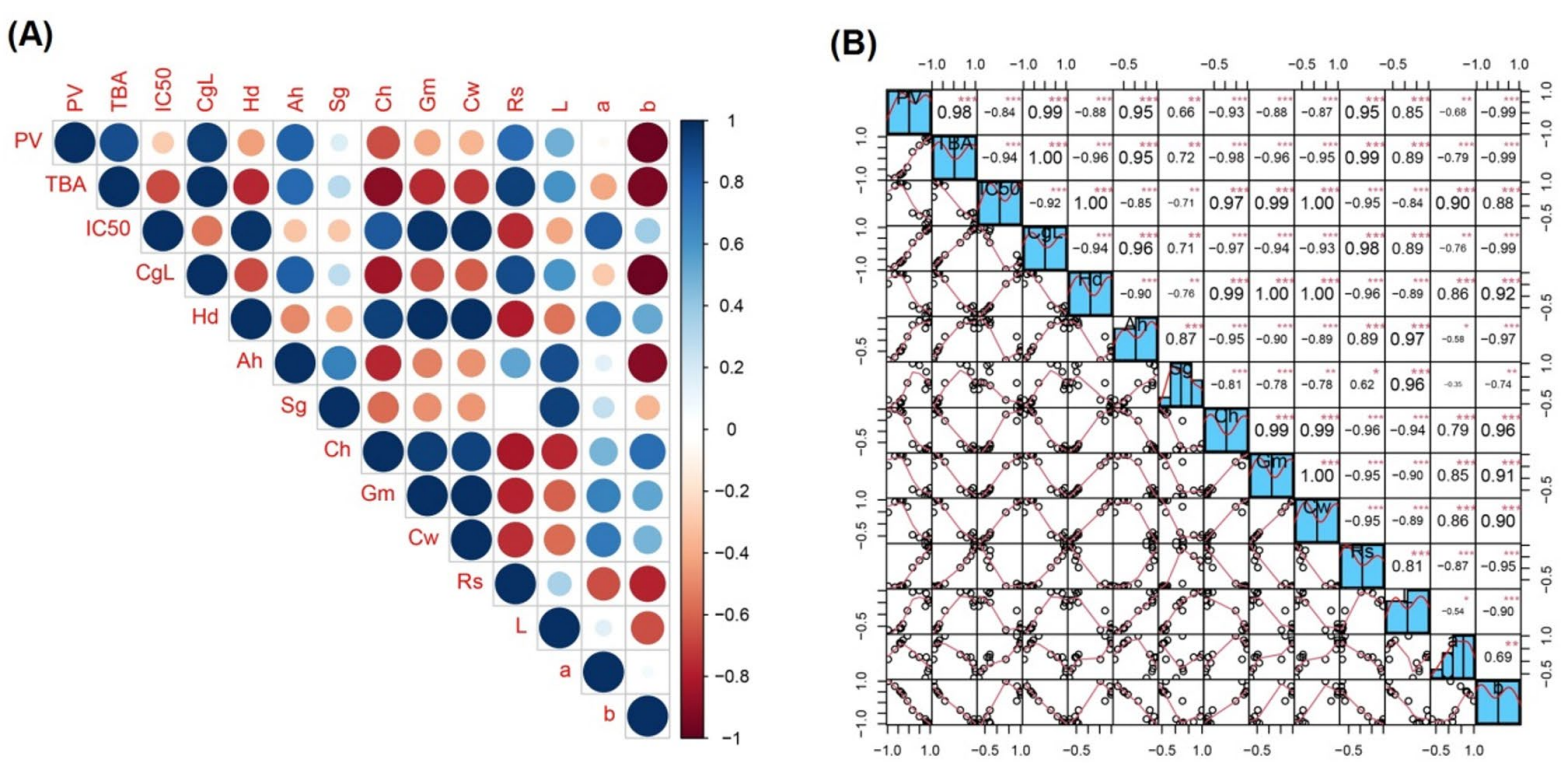
Quality control and correlation analysis of the data parameters in beef patty samples. (**A**) Pearson correlation analysis between samples. (**B**) Histogram Plot Analysis between samples. Color intensity indicates the strength of correlation, with deeper colors reflecting stronger correlations. Correlation coefficients significant at *p* ≤ 0.05. CgL: Cooking loss; Hd: Hardness; Ah: Adhesiveness; Sg: Springness; Ch: Cohesiveness; Gm: Gumminess; Cw: Chewiness ; Rs: Resilience ; L, a, b: color data.

Pearson correlation analysis on beef patties with varying additions of sacha inchi leaf extracts showed a significant value of < 0.05 for each variable, as presented in Fig. 2. This showed a relationship between the variables in beef patties containing sacha inchi leaf extracts with a very strong correlation level due to r value > 0.80. The correlation coefficient for peroxide and TBA value showed a very strong positive correlation. This showed a linear relationship between the variables, where higher formation of peroxide value in beef patties correlated with greater TBA value. In this study, peroxide value had a positive relationship with TBA because, during lipid oxidation, unsaturated fats were oxidized to form hydroperoxide compounds which were unstable and easily decomposed into more complex compounds such as MDA^37^.

The correlation coefficient of IC_50_ against TBA and peroxide value showed a very strong level with an r value > 0.80. This indicated a very strong positive correlation, where a higher IC50 value showed an elevated TBA and peroxide value. A lower IC_50_ value showed a stronger antioxidant activity, along with the formation of peroxide and TBA values. The stronger antioxidant activity as well as decrease in lipid oxidation was observed in beef patties. This was because sacha inchi leaf extracts were mostly composed of phenolic compounds and flavonoids with strong hydrogen donating activity or high radical scavenging capacity. Antioxidants can prevent the formation of free radicals and reactive oxygen species^38^.

### Effects of different concentrations of sacha inchi leaf extracts on lipidomic profiles of beef patties

Generally, lipids play an essential role in human physiology contributing significantly to the sensory attributes of food including flavor, texture, aroma, and consumer acceptance. Specific lipid molecules namely n-3/6 fatty acids, triacylglycerols (TAG), phospholipids (PL), and long-chain polyunsaturated fatty acids (LC-PUFA) are essential for human health, reducing the risk of cardiovascular diseases and inflammatory conditions such as asthma, and rheumatoid arthritis^39^. However, these lipids are susceptible to oxidation, which is influenced by temperature and heating time, capable of affecting lipid content and chemical composition. Lipidomics, a comprehensive method using LC-MS, can be applied to assess lipid profiles and their chemical composition^7^.

#### Untargeted lipidomics-phospholipid

Lipidomics analysis found 1000 different lipid species in the samples. This was possible because lipidomics methods were very accurate and selective, able to detect many lipids and sublipids^40^. After that, the lipids from all samples were analyzed to find compounds that were statistically significant (*p* < 0.05) and for further chemometric analysis. Chemometrics is useful for grouping or classifying data by their characteristics. PCA and PLS-DA are common methods used to classify samples^41^.

Lipidomics results showed that 350 lipids were significantly detected in positive ion mode (ESI+), while only 72 were found in negative ion mode (ESI^−^), as presented in Table 2. The total ion chromatogram (TIC) from all samples can be found on Supplementary figure S1-S4. The higher number of lipids detected in ESI^+^ could be attributed to the polarity of the compounds. Generally, positively charged ions are more polar and amenable to detection compared to negatively charged ions^18^.

**Table 2:** Significant lipid from lipidomics analysis in beef patties samples.

No	Sample	Lipid total	Lipid in ESI^+^	Lipid in ESI^−^	Significant	
					ESI^+^	ESI^−^
1	SI0	630	589	41	350	72
2	SI1	695	645	50		
3	SI2	905	810	95		
4	SI3	1274	1104	170		

SI0 = Beef Patty + SI 0%; SI1 = Beef Patty + SI 0,5%; SI2 = Beef Patty + SI 1%; SI3 = Beef Patty + SI 1,5%; SI = Sacha Inchi.

**Fig. 3:**
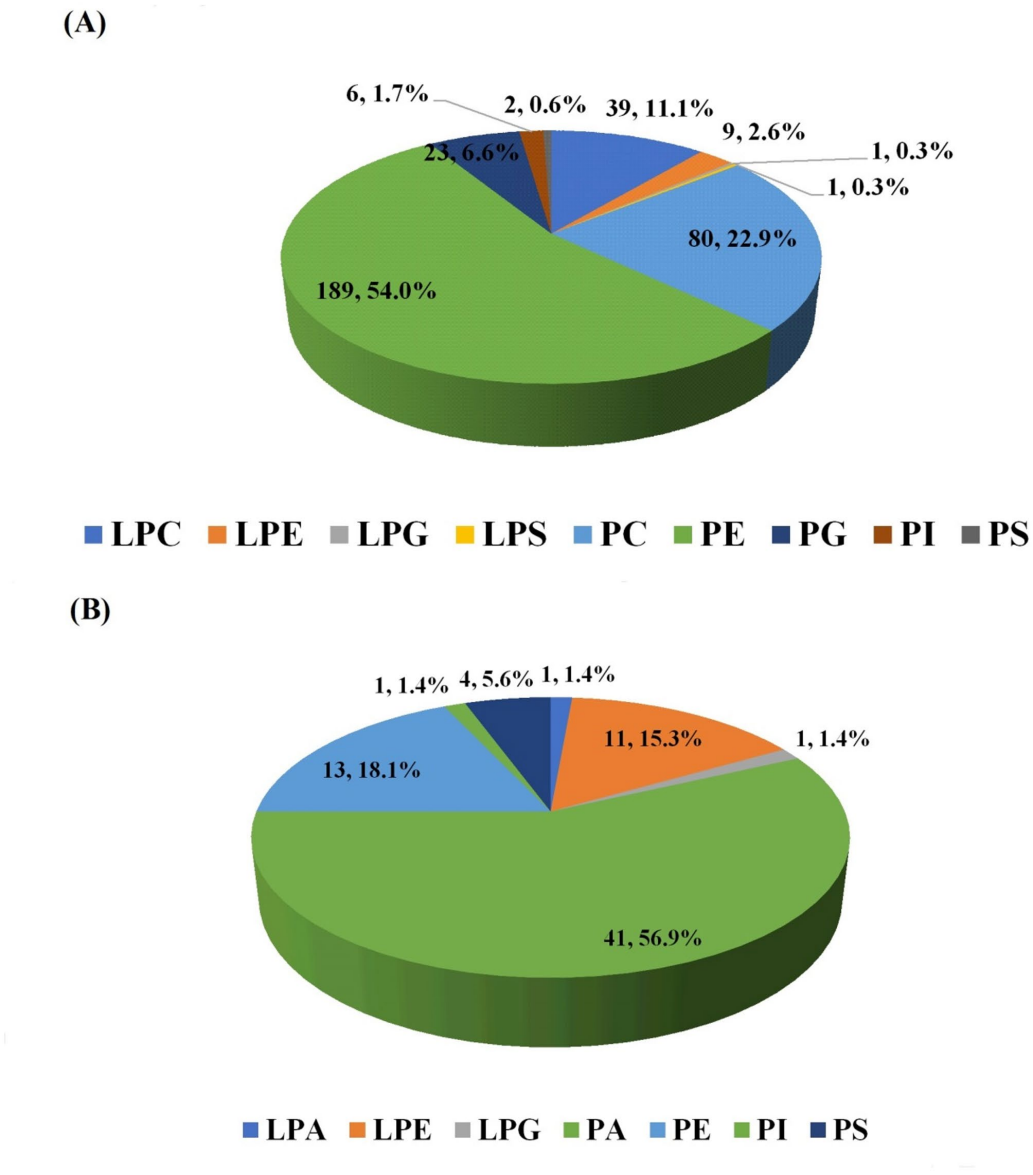
Glycerophospholipid subclass composition detected in beef patties with different concentrations of sacha inchi leaf extracts analyzed by UHPLC-HRMS. Panel (**A**) presents positive ion mode (ESI+), and panel (**B**) negative ion mode (ESI-). Data illustrate the number of lipid species identified within each subclass: LPC (lysophosphatidylcholine), LPE (lysophosphatidylethanolamine), PC (phosphatidylcholine), PE (phosphatidylethanolamine), LPG (lysophosphatidylglycerol), LPS (lysophosphatidylserine), PG (phosphatidylglycerol), PI (phosphatidylinositol), PS (phosphatidylserine), LPA (lysophosphatidic acid), and PA (phosphatidic acid).

Based on the lipid profiles obtained from both ESI^+^ and ESI^−^ modes in the samples, the detected lipids were primarily categorized as glycerophospholipids, as shown in Fig. 3. Lipids can be classified into eight categories based on their function, chemical properties, and characteristics. These include fatty acids, glycerolipids, glycerophospholipids, sphingolipids, sterol, prenol, saccharolipids, and polyketides^40^. TAG and phospholipids are the predominant lipids found in meat. According to Maritha et al.^18^glycerophospholipids are the most abundant class in beef and pork due to their important hydrogen bonding for phospholipid membranes. This high abundance can be attributed to the essential role of phospholipids in contributing to the flavor and texture of meat products. Furthermore, phospholipids contain polyunsaturated fatty acids, which can more readily participate in Maillard reactions^42^.

Based on Fig. 3A, ESI^+^ analysis of the samples detected lipids belonging to several subclasses. These included PE (Phosphatidylethanolamine), PC (Phosphatidylcholine), LPC (Lysophosphatidylcholine), PG (Phosphatidylglycerol), LPE (Lysophosphatidylethanolamine), PI (Phosphatidylinositol), PS (Phosphatidylserine), LPG (Lysophosphatidylglycerol), and LPS (Lysophosphatidylserine). The results showed that PE was the most abundant subclass in ESI^+^, with 189 lipids detected. ESI^−^ analysis also detected several subclasses, namely PA (Phosphatidic Acid), PE, LPE, PS, LPA (Lysophosphatidic acid), LPG, and PI. The most abundant subclass in ESI^−^ was PA, with 41 lipids detected. The high abundance of PE in ESI + was due to its prevalence in glycerophospholipids. The major subclasses of glycerophospholipids included PC, PE, PI, and PS. Moreover, PC, PE, LPC, and LPE are commonly found in meat^43^. The high abundance of PA in ESI^−^ can be attributed to the fact that this subclass frequently forms [M – H] – anions^44^.

Based on lipidomics results, six lipids had the highest compositions in the control beef patties including PE(10:0p/11:0) + H, PE(12:0p/9:0) + H, PE(4:0/16:1) + H, PE(6:0/14:1) + H, PE(8:0e/13:1) + H, and PE(8:0p/13:0) + H in ESI^+^, and LPE(22:5)-H in ESI^−^. In beef patties with 0.5% sacha inchi leaf extracts, LPC(18:1) + H was the most abundant lipid in ESI^+^, while LPE(18:2)-H dominated in ESI^−^. Beef patties with 1% extracts had four major lipids in ESI^+^, namely LPC(18:1) + H, PE(8:0p/13:0) + H, PC(4:0/13:1) + H, and PC(8:0p/10:0) + H, and LPE(18:2)-H in ESI^−^. Samples with 1.5% extracts had six major lipids in ESI^+^, including PE(8:0p/13:0) + H, PE(4:0/16:1) + H, PE(10:0p/11:0) + H, PE(12:0p/9:0) + H, PE(6:0/14:1) + H, and PE(8:0e/13:1) + H, and LPE(18:2)-H in ESI^−^. Additionally, 21 lipids (8 in ESI^+^ and 13 in ESI^−^) were exclusively detected in beef patties with added sacha inchi extracts, but not in the control. These included PE(15:0/8:0)-H, LPG(25:1)-H, PE(4:0/13:0)-H, PE(8:0e/10:0)-H, LPE(22:6)-H, PI(34:0/18:1)-H, PE(8:0e/15:1)-H, LPG(25:1) + H, LPC(12:0e) + H, LPC(14:0e) + H, PC(8:0p/9:0) + CH3COO, PG(11:0/14:1) + H, LPC(17:3) + H, LPC(18:2p) + H, LPC(20:1) + H, LPC(17:2) + H, LPC(18:2e) + H, LPC(18:1p) + H, LPE(20:2) + H, and PE(4:0/15:2) + H. The most abundant lipids in each sample are summarized in the Table 3.

**Table 3:** Major glycerophospholipids composition in beef patties samples.

Lipid ion	Sample			
	SI0	SI1	SI2	SI3
ESI^+^	PE(10:0p/11:0) + H PE(12:0p/9:0) + H PE(4:0/16:1) + H PE(6:0/14:1) + H PE(8:0e/13:1) + H PE(8:0p/13:0) + H	LPC(18:1) + H	LPC(18:1) + H PE(8:0p/13:0) + H PC(4:0/13:1) + H PC(8:0p/10:0) + H	PE(8:0p/13:0) + H PE(4:0/16:1) + H PE(10:0p/11:0) + H PE(12:0p/9:0) + H PE(6:0/14:1) + H PE(8:0e/13:1) + H
ESI^−^	LPE(22:5)-H	LPE(18:2)-H	LPE(18:2)-H	LPE(18:2)-H

SI0 = Beef Patty + SI 0%; SI1 = Beef Patty + SI 0,5%; SI2 = Beef Patty + SI 1%; SI3 = Beef Patty + SI 1,5%; SI = Sacha Inchi.

LPC (*Lyso-Phosphatidyl Choline*); PC (*Phospatidyl Choline*); PE (*Phospatidyl Ethanolamine*); LPE (*Lyso-Phospatidyl Ethanolamine*).

According to Correia et al.^45^glycerophospholipids play a significant role in human health. Several subclasses of glycerophospholipids have specific health benefits, such as PE, which is beneficial in hepatic steatosis. PC is related to human brain function, a reduced risk of type 2 diabetes, and has been implicated in heart, skeletal muscle, liver health, and metabolism^46^. The high abundance of PE and PC in beef patties suggested potential health benefits due to their unsaturated fatty acid content and potent antioxidant properties. These lipid compositions contributed to cardiovascular health by enhancing the ability of HDL (High-density Lipoprotein) to protect vascular cells from damage^47^. LPE was reported to show anti-inflammatory and anti-hemostatic effects, while LPG and LPS were included in immunity and angiogenesis^48^.

#### Chemometric analysis

**Fig. 4:**
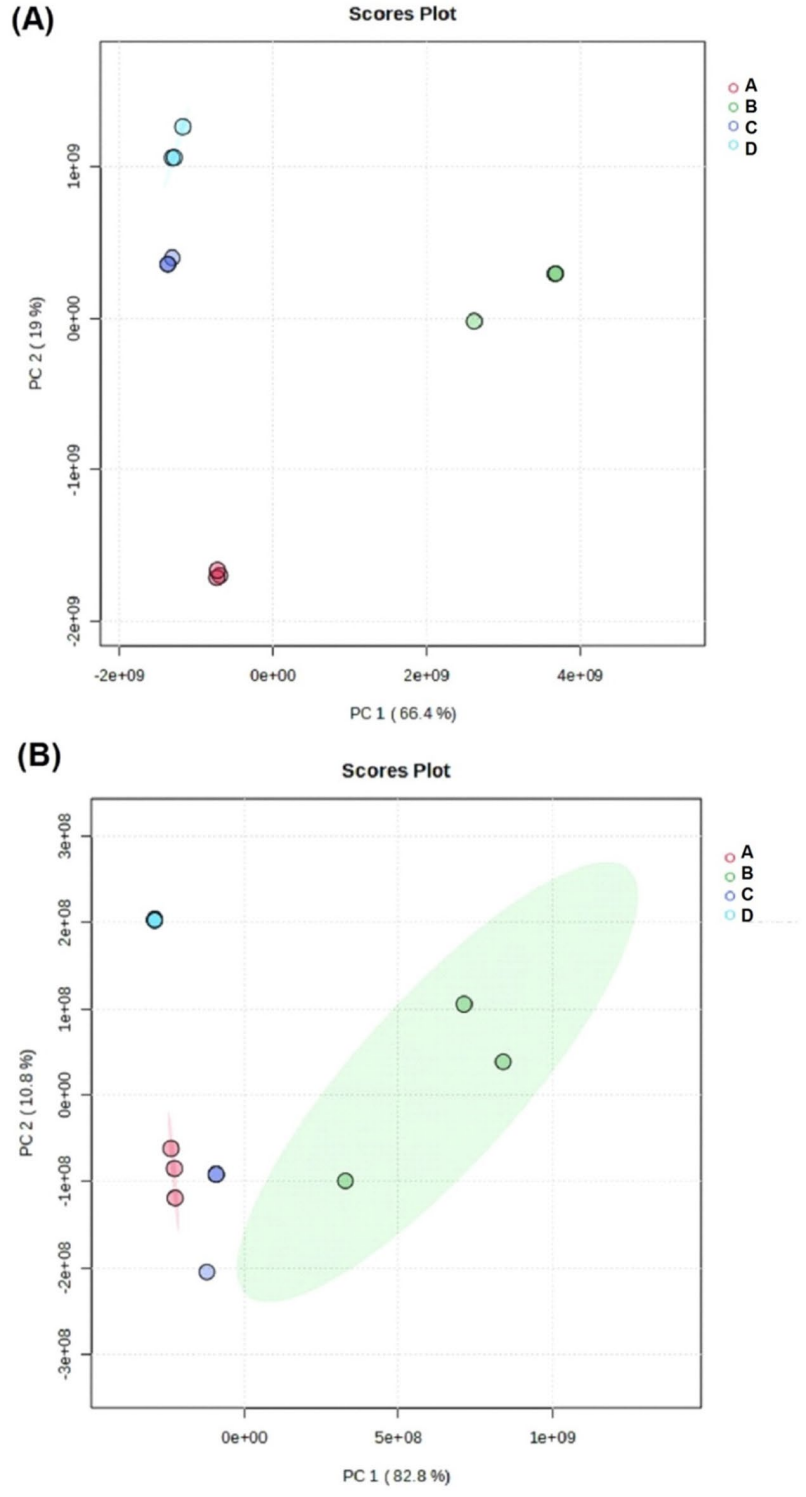
Principal component analysis (PCA) score plots of lipidomic profiles in beef patties supplemented with sacha inchi leaf extracts: (**A**) positive ionization (ESI+) mode, (**B**) negative ionization (ESI^−^) mode. Sample groups: Control (**D**; no extract), SI 0.5% (A; 0.5% extract), SI 1% (C; 1.0% extract), and SI 1.5% (**B**; 1.5% extract), SI: Sacha Inchi. Each point represents an independent replicate, demonstrating clear separation based on extract concentration.

Chemometric analysis, specifically PCA was used to identify differences in lipid profiles among the samples. PCA is a tool that can effectively show relationships between objects in a dataset^49^. The results of PCA for both ESI^+^ and ESI^−^ modes are presented in Fig. 4, respectively. These figures show the interpretation of lipid profiles in beef patties with varying concentrations of sacha inchi leaf extracts. The PCA scores plot in Fig. 3 showed that the lipid profiles of the four samples (control and three treatment groups) in ESI^+^ mode were distinct from each other. This suggested that the addition of sacha inchi leaf extracts at different concentrations significantly altered the lipid composition of beef patties. Similarly, the PCA scores plot in ESI^−^ mode showed distinct clustering of samples, suggesting that the lipid profiles were influenced by extracts concentration.

PLS-DA was used to further differentiate between sample groups based on their lipid profiles. Compared to PCA, PLS-DA incorporates class information, allowing for more robust classification between groups. Consequently, PLS-DA can be used to identify the most influential lipids in distinguishing the samples^50^. The variable importance in projection (VIP) scores from the PLS-DA model is also applicable to identify the lipids that contribute most to the separation between groups. VIP scores greater than 1 show that the corresponding lipid is a significant contributor to the model. The loading plots, represented by a color scale ranging from red (high concentration) to blue (low concentration), show the relative contribution of each lipid to the separation between groups^51^.

**Fig. 5:**
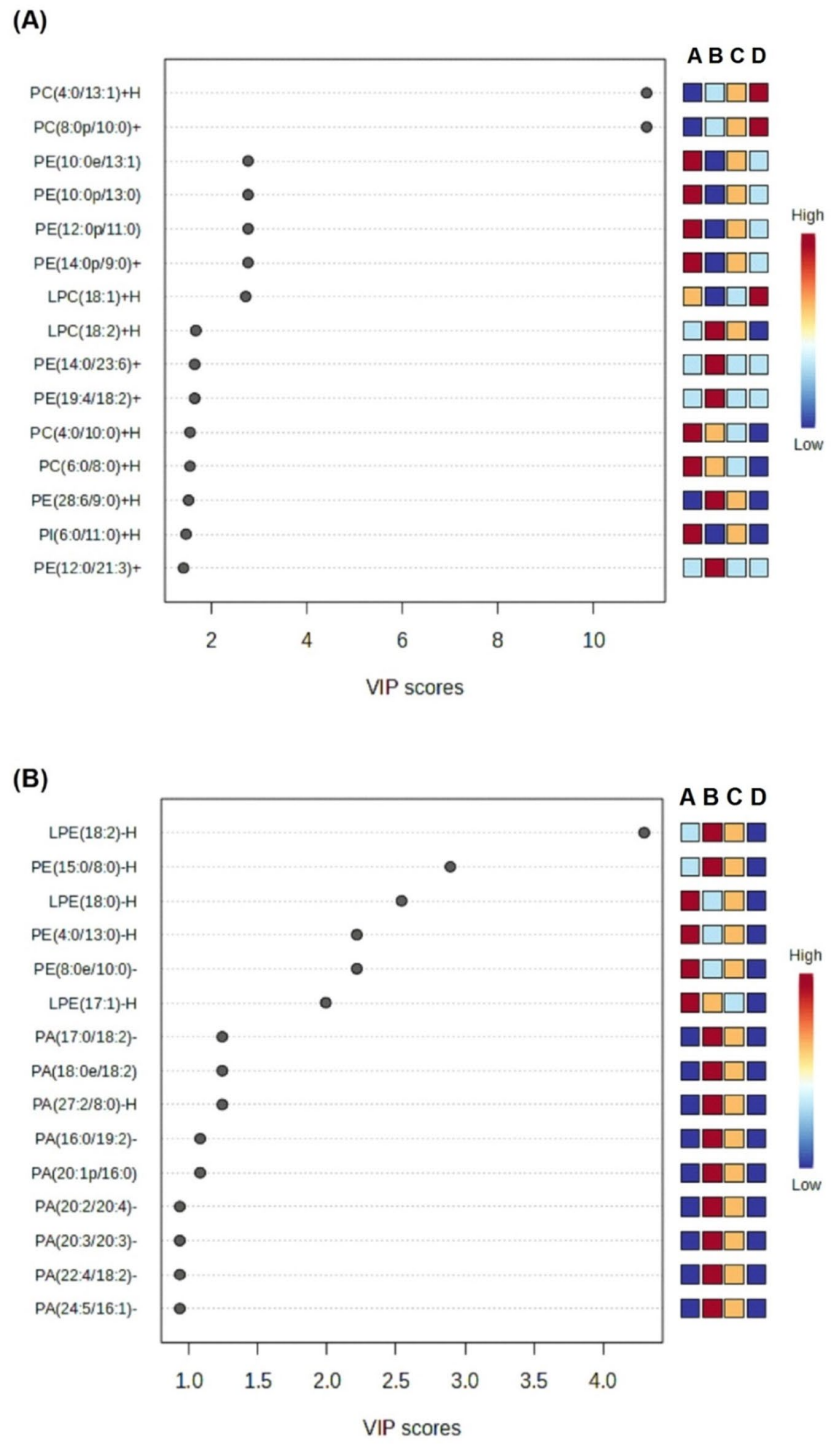
VIP scores from Partial Least Squares-Discriminant Analysis (PLS-DA) identifying lipid biomarkers significantly contributing to differences among beef patties supplemented with varying concentrations of sacha inchi leaf extracts. Panel (**A**) shows VIP scores for lipids detected in positive ion mode (ESI+), and panel (**B**) negative ion mode (ESI-). Lipids with VIP scores above 1.0 (marked by the red dashed line) were considered significant discriminants. A: Beef Patty + SI 0.5%; B: Beef Patty + SI 1.5%; C: Beef Patty + SI 1%; D: Control; SI: Sacha Inchi.

PLS-DA analysis of the ESI^+^ data showed 15 lipids with VIP scores greater than 1. This indicated that the lipids were significant in differentiating between beef patties, as shown in Fig. 5. These lipids were PC(4:0/13:1) + H, PC(8:0p/10:0) + H, PE(10:0e/13:1) + H, PE(10p:0/13:0) + H, PE(12:0p/11:0) + H, PE(14:0p/9:0) + H, LPC(18:1) + H, LPC(18:2) + H, PE(14:0/23:6) + H, PE(19:4/18:2) + H, PC(4:0/10:0) + H, PE(6:0/8:0) + H, PE(28:6/9:0) + H, PI(6:0/11:0) + H, and PE(12:0/21:3) + H. The control beef patties had the highest concentrations of three lipids, namely PC(4:0/13:1) + H, PC(8:0p/10:0) + H, and LPC(18:1) + H. The 1.5% sacha inchi extracts group had the highest concentrations of five lipids, namely LPC(18:2) + H, PE(14:0/23:6) + H, PE(19:4/18:2) + H, PE(28:6/9:0) + H, and PE(12:0/21:3) + H. The 0.5% extracts group had high concentrations of seven lipids, including PE(10:0e/13:1) + H, PE(10p:0/13:0) + H, PE(12:0p/11:0) + H, PE(14:0p/9:0) + H, PC(4:0/10:0) + H, PE(6:0/8:0) + H, and PI(6:0/11:0) + H. However, 1% extracts group did not show any lipids with extremely high concentrations but had moderate levels of nine lipids, namely PC(4:0/13:1) + H, PC(8:0p/10:0) + H, PE(10:0e/13:1) + H, PE(10p:0/13:0) + H, PE(12:0p/11:0) + H, PE(14:0p/9:0) + H, LPC(18:2) + H, PE(28:6/9:0) + H, and PI(6:0/11:0) + H.

PLS-DA analysis of the ESI^−^ data showed 11 lipids with VIP scores greater than 1. The results suggested that these lipids were significant in differentiating between beef patties. These lipids were LPE(18:2)-H, PE(15:0/18:0)-H, LPE(18:0)-H, PE(4:0/13:0)-H, PE(8:0e/10:0)-H, LPE(17:1)-H, PA(17:0/18:2)-H, PA(18:0e/18:2)-H, PA(27:2/8:0), PA(16:0/19:2)-H, and PA(20:1p/16:0)-H. The 1.5% sacha inchi extracts group had the highest concentrations of 11 lipids, namely LPE(18:2)-H, PE(15:0/8:0)-H, PA(17:0/18:2)-H, PA(18:0e/18:2)-H, PA(27:2/8:0), PA(16:0/19:2)-H, PA(20:1p/16:0)-H, PA(20:2/20:4)-H, PA(20:3/20:3)-H, PA(22:4/18:2)-H, and PA(24:5/16:1)-H. The 0.5% extracts group had high concentrations of four lipids, namely LPE(18:0)-H, PE(4:0/13:0)-H, PE(8:0e/10:0)-H, and LPE(17:1)-H. The control group indicated low concentrations of all lipids, while the 1% extracts group showed moderate concentrations of all lipids.

The lipidomics results revealed significant alterations in specific phospholipids following the incorporation of sacha inchi leaf extracts. Notably, from the PLS-DA results showed that beef patties with the highest concentration of sacha inchi leaf extracts (1.5%) predominantly contained unsaturated fatty acids. However, as extract concentration decreased, a higher proportion of saturated fatty acids was observed. This suggested that the addition of antioxidants from sacha inchi leaf extracts could prevent lipid oxidation. Generally, antioxidants are known to inhibit or slow down oxidation of unsaturated fatty acids in lipid-rich foods such as meat. According to Paula et al.^52^the incorporation of antioxidants could also contribute to overall health benefits, while Haraf et al.^53^ reported that marinating meat with natural antioxidants such as kiwiberry increase the total content of unsaturated fatty acids. These unsaturated fatty acids are linked to many health benefits, such as lowering the risk of heart disease, diabetes, rheumatoid arthritis, and cancer. Briggs et al.^54^ stated that consuming foods high in unsaturated fatty acids can lower the risk of heart disease and cholesterol compared to diets with a lot of saturated fats. In this study, beef patties with 1.5% sacha inchi extracts had LPC(18:2), a type of lipid that can help lower heart disease risk^55^. Also, the LPE(18:2) might help with insulin sensitivity and reduce the risk of diabetes and heart disease^56^.

**Fig. 6:**
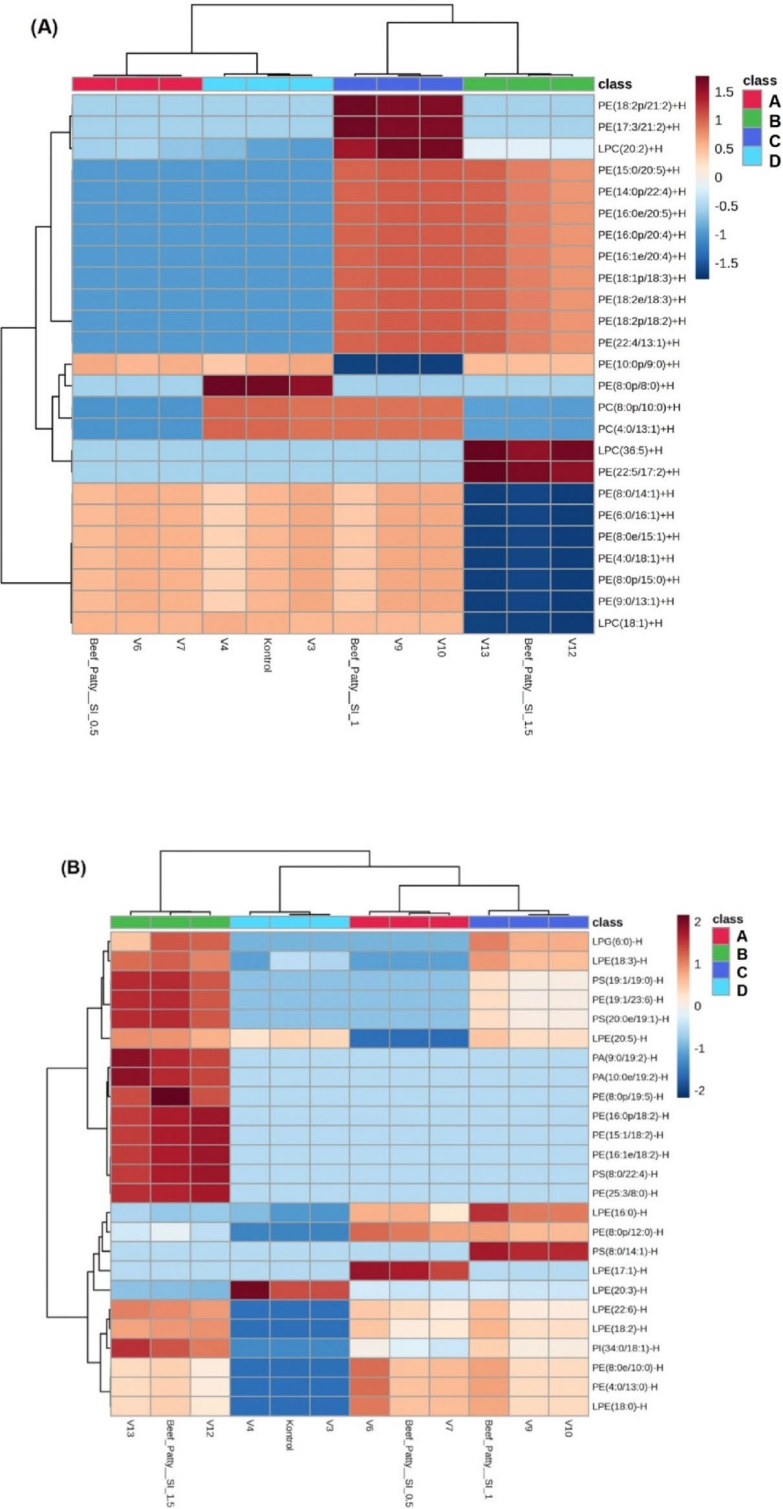
Heatmap clustering analysis of significantly altered lipid species in beef patties with different concentrations of sacha inchi leaf extracts in (**A**) positive ion (ESI+) and (**B**) negative ion (ESI-) modes. Color intensity indicates relative abundance, from blue (low) to red (high). Sample groups: Control (no extract), SI 0.5% (0.5% extract), SI 1% (1% extract), and SI 1.5% (1.5% extract). Data were analyzed using hierarchical clustering.

Lipid profiles in beef patties with added sacha inchi leaf extracts can be visualized using cluster analysis with a heatmap. This method helps show relationships between variables and is useful for analyzing the differences and similarities in the lipid profiles of the patties. Figure 6 shows the heatmap clustering of beef patties with different amounts of sacha inchi leaf extracts. Heatmap in Fig. 6A shows the lipid profile of beef patties with varying additions of sacha inchi leaf extracts in ESI^+^ mode. Samples with varying additions of sacha inchi leaf extracts are represented by different colors: light blue (without sacha inchi leaf extracts), pink (0.5% extracts), dark blue (1% extracts), and green (1.5% extracts). Heatmap results in ESI^+^ mode showed distinct clusters for each sample where beef patties with 1.5% sacha inchi leaf extracts had higher expression of LPC(36:5) + H and PE(22:5/17:2) + H lipids.

Figure 6B shows the lipid profile of beef patties with different amounts of sacha inchi leaf extracts in ESI- mode. The samples have different colors: light blue means no extract, pink is 0.5% extract, dark blue is 1%, and green is 1.5%. The heatmap in ESI- mode shows separate clusters for each sample. This shows that beef patties with different amounts of extract have different lipid profiles. Also, the heatmap shows that patties with 1.5% extract have more lipids overall compared to the others.

Despite extensive research on plant-derived antioxidants, very limited data are available on lipidomic responses in meat systems, particularly concerning novel sources like sacha inchi leaf extracts. Our study uniquely integrates advanced lipidomics techniques (UHPLC-HRMS) with traditional lipid oxidation assays (POV, TBA), providing comprehensive insights into the protective mechanisms at the molecular lipid level. Previous research primarily focused on general lipid oxidation parameters without detailed lipid subclass profiling^8,9^. Thus, our findings offer substantial novel insights into specific glycerophospholipid subclasses protected by sacha inchi leaf extracts, highlighting their potential nutritional and functional benefits in meat products. The identification of specific lipid biomarkers through chemometric analysis (PLS-DA and PCA) further enhances the novelty and applicability of our results. Future research might extend these findings by examining long-term stability and bioavailability of these preserved phospholipids in human dietary interventions.

### Limitations and future recommendations

Although our study clearly demonstrated antioxidant activity through the DPPH radical scavenging assay, we acknowledge that performing additional in vitro assays, such as ABTS, FRAP, or ORAC, would provide a more comprehensive characterization of antioxidant mechanisms. These assays could help further validate antioxidant capacity and better explain the protective effects observed against lipid oxidation. Future studies should explore these additional assays to fully elucidate the antioxidant profiles and bioactive substance contents of sacha inchi leaf extracts.

## Conclusion

In conclusion, the result showed that there was significant inhibition of lipid oxidation when sacha inchi leaf extracts were added to pan-fried beef patties at higher concentrations. The antioxidant activity was also improved with significant differences observed in lipid profiles based on varying concentrations. Based on VIP scores from PLS-DA, 15 compounds in ESI + and 11 in ESI- with VIP scores > 1 were identified as potential lipid biomarkers. Beef patties with 1.5% sacha inchi leaf extracts showed the most favorable lipid profile, characterized by the presence of LPC(18:2) + H, PE(14:0/23:6) + H, PE(19:4/18:2) + H, and PE(12:0/21:3) in ESI^+^ and LPE(18:2)-H, PE(15:0/8:0)-H, PA(17:0/18:2)-H, PA(18:0e/18:2)-H, PA(27:2/8:0), PA(16:0/19:2)-H, PA(20:1p/16:0)-H, PA(20:2/20:4)-H, PA(20:3/20:3)-H, PA(22:4/18:2)-H, and PA(24:5/16:1)-H in ESI^−^. This study suggested that the addition of 1.5% sacha inchi leaf extracts to beef patties had the potential to significantly improve health profiles. Moreover, prospective clinical studies were recommended to confirm the results and evaluate their impact on human health.

## Supplementary Information

Below is the link to the electronic supplementary material.

Supplementary Material 1

## Data Availability

Data availability statementThe data presented in this study are available on request from the corresponding author.

## References

[1] Onopiuk, A., Kołodziejczak, K., Marcinkowska-Lesiak, M. & Poltorak, A. Determination of polycyclic aromatic hydrocarbons using different extraction methods and HPLC-FLD detection in smoked and grilled meat products. *Food Chem.* **373**, 131506 (2022). 34758433 10.1016/j.foodchem.2021.131506

[2] Aoudeh, E., Oz, E. & Oz, F. Effect of beef patties fortification with black Garlic on the polycyclic aromatic hydrocarbons (PAHs) content and toxic potency. *Food Chem.* **428**, 136763 (2023). 37421662 10.1016/j.foodchem.2023.136763

[3] Beya, M. M., Netzel, M. E., Sultanbawa, Y., Smyth, H. & Hoffman, L. C. Kakadu Plum (Terminalia ferdinandiana) bioactivity against spoilage microorganisms and oxidative reactions in refrigerated Raw beef patties under modified atmosphere packaging. *Meat Sci.* **204**, 109268 (2023). 37379705 10.1016/j.meatsci.2023.109268

[4] Parvin, R., Seo, J., Eom, J. U., Ahamed, Z. & Yang, H. S. Inhibitory and antioxidative capacity of nutmeg extracts on reduction of lipid oxidation and heterocyclic amines in pan-roasted beef patties. *Meat Sci.* **197**, 109064 (2023). 36493554 10.1016/j.meatsci.2022.109064

[5] Li, J., Han, D., Huang, F. & Zhang, C. Effect of reheating methods on eating quality, oxidation and flavor characteristics of braised beef with potatoes dish. *Int. J. Gastronomy Food Sci.* **31**, 100659 (2023).

[6] Nuora, A. et al. The impact of beef steak thermal processing on lipid oxidation and postprandial inflammation related responses. *Food Chem.* **184**, 57–64 (2015). 25872426 10.1016/j.foodchem.2015.03.059

[7] Zhang, M. et al. Lipidomic profile changes of yellow-feathered chicken meat during thermal processing based on UPLC-ESI-MS approach. *Food Chem.* **399**, 133977 (2023). 35994857 10.1016/j.foodchem.2022.133977

[8] Hadidi, M. et al. Plant by-product antioxidants: control of protein-lipid oxidation in meat and meat products. *LWT* **169**, 114003 (2022).

[9] Macho-González, A. et al. Can meat and meat-Products induce oxidative stress?? *Antioxidants (Basel Switzerland)* 9 (2020). 10.3390/antiox9070638 PMC7402184 32698505

[10] Wang, D., Xiao, H., Lyu, X., Chen, H. & Wei, F. Lipid oxidation in food science and nutritional health: A comprehensive review. *Oil Crop Sci.* **8**, 35–44 (2023).

[11] Martini, S., Cattivelli, A., Conte, A. & Tagliazucchi, D. Black, green, and Pink pepper affect differently lipid oxidation during cooking and in vitro digestion of meat. *Food Chem.* **350**, 129246 (2021). 33610839 10.1016/j.foodchem.2021.129246

[12] Prommachart, R. et al. The effect of black rice water extract on surface color, lipid oxidation, microbial growth, and antioxidant activity of beef patties during chilled storage. *Meat Sci.* **164**, 108091 (2020). 32126446 10.1016/j.meatsci.2020.108091

[13] Xu, Y. et al. Inhibitory effect of mulberry leaf (*Morus alba L.*) extract on the formation of free and bound heterocyclic amines in pan-fried muscovy duck (Cairina moschata) patties. *Food Control*. **144**, 109359 (2023).

[14] Wilson, D. W. et al. The role of food antioxidants, benefits of functional foods, and influence of feeding habits on the health of the older person: an overview. *Antioxidants (Basel Switzerland)* 6 (2017). 10.3390/antiox6040081 PMC5745491 29143759

[15] S., M. Antioxidants as functional foods in health and diseases. *Austin J. Nutri Food Sci* **3**, 1067 (2015).

[16] Kittibunchakul, S. et al. Effects of maturity and thermal treatment on phenolic profiles and in vitro Health-Related properties of Sacha inchi leaves. *Plants (Basel Switzerland)* 11 (2022). 10.3390/plants11111515 PMC9182973 35684288

[17] Jia, W., Li, R., Wu, X., Liu, S. & Shi, L. UHPLC-Q-Orbitrap HRMS-based quantitative lipidomics reveals the chemical changes of phospholipids during thermal processing methods of Tan sheep meat. *Food Chem.* **360**, 130153 (2021). 34034056 10.1016/j.foodchem.2021.130153

[18] Maritha, V. et al. Lipidomics analysis for Halal authentication of triceps brachii, longissimus dorsi, and biceps femoris meats: profiling the lipid composition. *LWT* **185**, 115187 (2023).

[19] Zakaria, Z. et al. Phenolic compounds, free radical scavenging activity and α-Glucosidase Inhibition properties of green, oolong and black Sacha Inchi tea extract. *Curr. Res. Nutr. Food Sci. J.* **11**, 1127–1142 (2023).

[20] Khan, I. A. et al. Mitigation of heterocyclic amines by phenolic compounds in allspice and Perilla frutescens seed extract: the correlation between antioxidant capacities and mitigating activities. *Food Chem.* **368**, 130845 (2022). 34419791 10.1016/j.foodchem.2021.130845

[21] Gulcin, İ. & Alwasel, S. H. DPPH radical scavenging assay. *Processes* **11**, 2248 (2023).

[22] Harlina, P. W., Yuliana, T., Fetriyuna, Shahzad, R. & Ma, M. Study on the development and functional characteristics of salted egg with liquid smoke. *Food Sci. Anim. Resour.* **43**, 471–490 (2023). 37181222 10.5851/kosfa.2023.e10 PMC10172823

[23] Sallam, K. I., Ishioroshi, M. & Samejima, K. Antioxidant and antimicrobial effects of Garlic in chicken sausage. *Lebensmittel-Wissenschaft + [i E Und] Technologie Food Sci. + Technol. Sci. + technologie Alimentaire*. **37**, 849–855 (2004). 10.1016/j.lwt.2004.04.001 PMC1805705 17330154

[24] Oparaku, N. F., Mgibenka, B. & Eyo, J. E. Proximate and organoleptic characteristics of sun and solar dried fish. *Anim. Res. Int.* **7**, 1169–1175 (2010).

[25] Harlina, P. W., Ma, M. & Shahzad, R. Quantification of lipidomics profiling using UPLC-QE-HESI- lipid analysis on the salted Duck egg incorporated with clove extract. *Eur. J. Lipid Sci. Technol.* **123**, 2000284 (2021).

[26] Mabrouki, S. et al. Texture profile analysis of homogenized meat and plant-based patties. *Int. J. Food Prop.* **26** (2), 2757–2771 (2023).

[27] Sánchez, C. N. et al. Analysis of beef quality according to color changes using computer vision and White-Box machine learning techniques. *Heliyon* **9**(7) (2023). 10.1016/j.heliyon.2023.e17976 PMC10375562 37519729

[28] Abeyrathne, E., Nam, K. & Ahn, D. U. Analytical methods for lipid oxidation and antioxidant capacity in food systems. *Antioxidants (Basel Switzerland)* **10** (2021). 10.3390/antiox10101587 PMC8533275 34679722

[29] Rahman, M. H., Hossain, M. M., Rahman, S. M., Amin, M. R. & Oh, D. H. Evaluation of physicochemical deterioration and lipid oxidation of beef muscle affected by Freeze-thaw cycles. *Korean J. Food Sci. Anim. Resour.* **35**, 772–782 (2015). 26877637 10.5851/kosfa.2015.35.6.772 PMC4726957

[30] Zhang, Y. et al. Understanding beef flavour and overall liking traits using two different methods for determination of thiobarbituric acid reactive substance (TBARS). *Meat Sci.* **149**, 114–119 (2019). 30502609 10.1016/j.meatsci.2018.11.018

[31] Del Rio, D. et al. A review of recent studies on malondialdehyde as toxic molecule and biological marker of oxidative stress. *Nutr. Metabolism Cardiovasc. Dis.* **15** (4), 316–328 (2005). 10.1016/j.numecd.2005.05.003 16054557

[32] Ma, L. et al. Food matrixes play a key role in the distribution of contaminants of lipid origin: A case study of malondialdehyde formation in vegetable oils during deep-frying. *Food Chem.* **347**, 129080 (2021). 33508586 10.1016/j.foodchem.2021.129080

[33] Wuttisin, N., Nararatwanchai, T. & Sarikaputi, A. Total phenolic, flavonoid, flavonol contents and antioxidant activity of Inca peanut (Plukenetia volubilis L.) leaves extracts. *Food Res.* **5**, 216–224 (2020).

[34] Choe, E. & Min, D. B. Mechanisms of antioxidants in the oxidation of foods. *Compr. Rev. Food Sci. Food Saf.* **8**, 345–358 (2009).

[35] Kiptiyah, S. Y., Santoso, U., Harmayani, E. & Supriyadi & Effect of heat treatment on antioxidant and antibacterial activity of *Kaempferia galanga L.* extract. *Food Res.* **7**, 205–213 (2023).

[36] Hihat, S., Remini, H. & Madani, K. Effect of oven and microwave drying on phenolic compounds and antioxidant capacity of coriander leaves. *Int. Food Res. J.* **24**, 503–509 (2017).

[37] Gheisari, H. R. Correlation between acid, TBA, peroxide and iodine values, catalase and glutathione peroxidase activities of chicken, cattle and camel meat during refrigerated storage. *Vet. World*. **4**, 153–157 (2011).

[38] Amaral, A. B., Silva, M. V. & Lannes, S. C. d. S. Lipid oxidation in meat: mechanisms and protective factors – a review. *Food Sci. Technol.* **38**, 1–15 (2018).

[39] Martakos, I. C., Tzavellas, I. F., Dasenaki, M. E. & Thomaidis, N. S. Food lipidomics: development and application of a cutting-edge untargeted 4D HRMS workflow for the lipid profiling of food of animal origin. *J. Food Compos. Anal.* **131**, 106232 (2024).

[40] Wu, Z., Bagarolo, G. I., Thoröe-Boveleth, S. & Jankowski, J. Lipidomics: mass spectrometric and chemometric analyses of lipids. *Adv. Drug Deliv Rev.* **159**, 294–307 (2020). 32553782 10.1016/j.addr.2020.06.009

[41] González-Domínguez, R. & Sayago, A. & Fernández-Recamales, Á. An overview on the application of chemometrics tools in food authenticity and traceability. *Foods (Basel Switzerland)* **11** (2022). 10.3390/foods11233940 PMC9738746 36496748

[42] Li, C., Al-Dalali, S., Zhou, H., Wang, Z. & Xu, B. Influence of mixture of spices on phospholipid molecules during water-boiled salted Duck processing based on shotgun lipidomics. *Food Res. Int.* **149**, 110651 (2021). 34600653 10.1016/j.foodres.2021.110651

[43] Harlina, P. W. et al. Possibilities of liquid chromatography mass spectrometry (LC-MS)-Based metabolomics and lipidomics in the authentication of meat products: A mini review. *Food Sci. Anim. Resour.* **42**, 744–761 (2022). 36133639 10.5851/kosfa.2022.e37 PMC9478982

[44] Randolph, C. E., Blanksby, S. J. & McLuckey, S. A. Enhancing detection and characterization of lipids using charge manipulation in electrospray ionization-tandem mass spectrometry. *Chem. Phys. Lipids*. **232**, 104970 (2020). 32890498 10.1016/j.chemphyslip.2020.104970 PMC7606777

[45] Correia, B. et al. Evaluation of the Amazon river seasonal influences on glycerophospholipids in wild fish. *J. Braz. Chem. Soc.* (2024).

[46] Zhou, X. et al. Glycerophospholipids in sea cucumber (Stichopus japonicus) and its processing by-products serve as bioactives and functional food ingredients. *J. Food Bioactives*. **1**, 134–142 (2018).

[47] Chen, H. et al. Comprehensive metabolomics identified the prominent role of glycerophospholipid metabolism in coronary artery disease progression. *Front. Mol. Biosci.* **8**, 632950 (2021). 33937325 10.3389/fmolb.2021.632950 PMC8080796

[48] Tan, S. T., Ramesh, T. & Toh, X. R. Emerging roles of lysophospholipids in health and disease. *Prog. Lipid Res.* **80**, 101068 (2020). 33068601 10.1016/j.plipres.2020.101068

[49] Shin, E. C. et al. Chemometric approach to fatty acid profiles in soybean cultivars by principal component analysis (PCA). *Prev. Nutr. Food Sci.* **17**, 184–191 (2012). 24471082 10.3746/pnf.2012.17.3.184 PMC3866742

[50] Ruiz-Perez, D., Guan, H., Madhivanan, P., Mathee, K. & Narasimhan, G. So you think you can PLS-DA? *BMC Bioinform.* **21**, 2 (2020). 10.1186/s12859-019-3310-7 PMC7724830 33297937

[51] Mashiane, P. et al. Cooking African pumpkin leaves (Momordica balsamina L.) by Stir-Frying improved bioactivity and bioaccessibility of Metabolites—Metabolomic and chemometric approaches. *Foods (Basel Switzerland)*. **10**, 2890 (2021). 34829171 10.3390/foods10112890 PMC8621757

[52] Fernandes, R. P. P., Trindade, M. A. & de Melo, M. P. in *Alternative and Replacement Foods* (eds Alina Maria Holban & Alexandru Mihai Grumezescu) 31–64 (Academic Press) (2018).

[53] Haraf, G., Goluch, Z., Teleszko, M. & Latocha, P. Antioxidant activity and fatty acid profile of Sous-Vide beef marinated with Kiwiberry fruit pulp: effects of level addition and refrigerated storage. *Foods (Basel Switzerland)*. **13**, 1446 (2024). 38790746 10.3390/foods13101446 PMC11120118

[54] Briggs, M. A., Petersen, K. S. & Kris-Etherton, P. M. Saturated fatty acids and cardiovascular disease: replacements for saturated fat to reduce cardiovascular risk. *Healthcare (Basel Switzerland)* 5 (2017). 10.3390/healthcare5020029 PMC5492032 28635680

[55] Gonzalez-Freire, M. et al. Targeted metabolomics shows low plasma lysophosphatidylcholine 18:2 predicts greater decline of gait speed in older adults: the Baltimore longitudinal study of aging. *J. Gerontol. Ser. A*. **74**, 62–67 (2018). 10.1093/gerona/gly100 PMC6298185 29788121

[56] Cho, Y. K. et al. Lipid remodeling of adipose tissue in metabolic health and disease. *Exp. Mol. Med.* **55**, 1955–1973 (2023). 37653032 10.1038/s12276-023-01071-4 PMC10545718

