# Supplementary material for: Lipidomics analysis of phospholipid profiles and oxidative stability in pan-fried beef patties incorporating sacha inchi leaf extracts
Source: Sci Rep. 2025 Aug 2;15:28233. doi: 10.1038/s41598-025-13267-x (PMC12317974; doi:10.1038/s41598-025-13267-x)
Supplement: Supplementary file 1 — Supplementary Material 1 [file 41598_2025_13267_MOESM1_ESM.docx]

**Supplementary Files**

**Table S1. MS Conditions**

| **Full MS** | | |
| --- | --- | --- |
| Resolution | : | 70,000 |
| AGC Target | : | 3e6 |
| Maximum IT | : | 100 ms |
| Scan Range | : | 100 to 1500 m/z |
| **dd-MS*/dd-SIM** | | |
| Resolution | : | 17,500 |
| AGC Target | : | 1e5 |
| Maximum IT | : | 50 ms |
| Loop count | : | 5 |
| TopN | : | 5 |
| Isolation window | : | 4.0 m/z |
| Fixed first mass | : | - |
| (N)CE/stepped (N)CE nce | : | 18, 35, 53 |
| **dd Settings** | | |
| Minimum AGC Target | : | 8,00e3 |
| Intensity thereshold | : | 1.6e5 |
| Apex trigger | : | - |
| Charge exclusion | : | - |
| Peptide match | : | Prefered |
| Exclude isotopes | : | On |
| Dynamic exclusion | : | 10.0 s |

| **HESI Source** | | |
| --- | --- | --- |
| **Actual** | | |
| Sheath gas flow rate | 15 | 0 |
| Aux gas flow rate | 3 | 0 |
| Sweep gas flow rate | 0 | 0 |
| Spray voltage ([kV]) | 3.80 | 0.00 |
| Spray current (µA) |  | 0.10 |
| Capillary temp (°C) | 320 | 320 |
| S-lens RF Level | 50.0 |  |
| Aux gas heater temp (°C) | 0 | 39 |

**Figure S1. Total Ion Chromatogram (TIC): Control Samples**

| 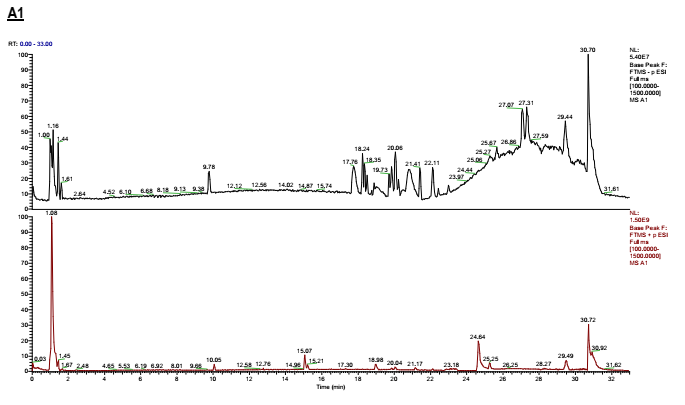 |
| --- |
| 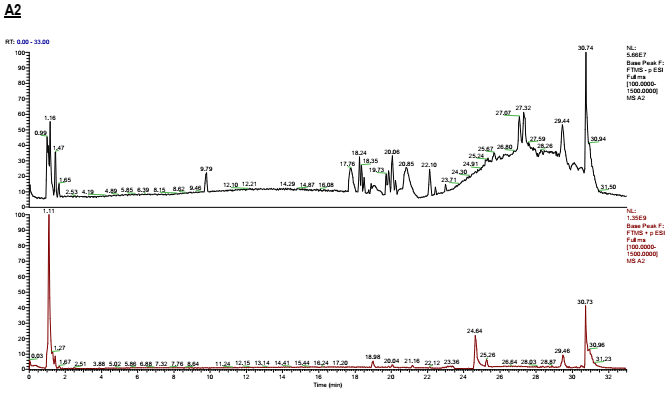 |
| 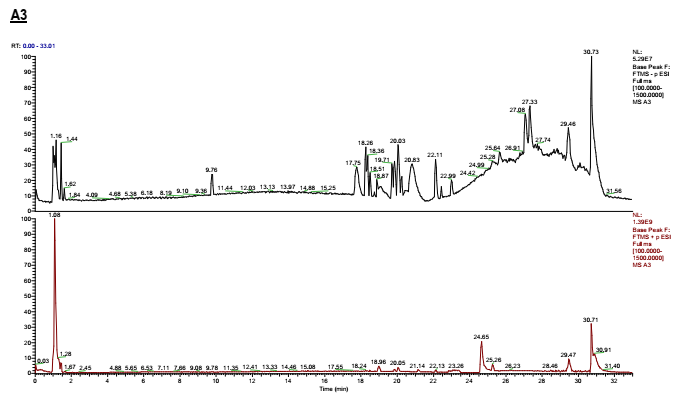 |

**Figure S2. Total Ion Chromatogram (TIC): Beef Patty 0.5% Sacha Inchi leaf extracts**

| 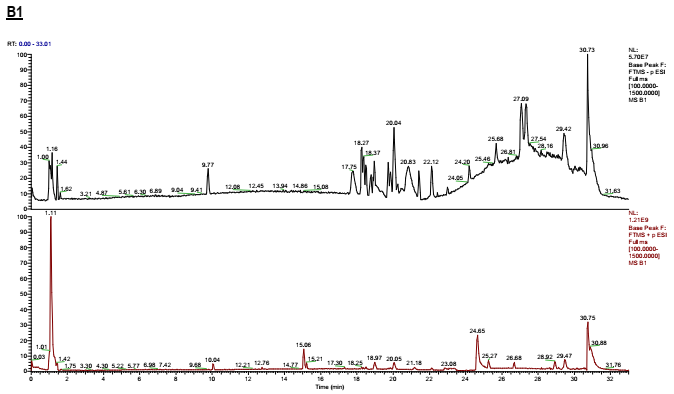 |
| --- |
| 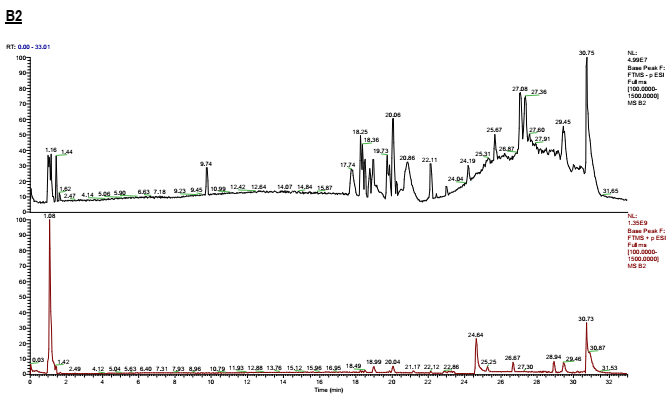 |
| 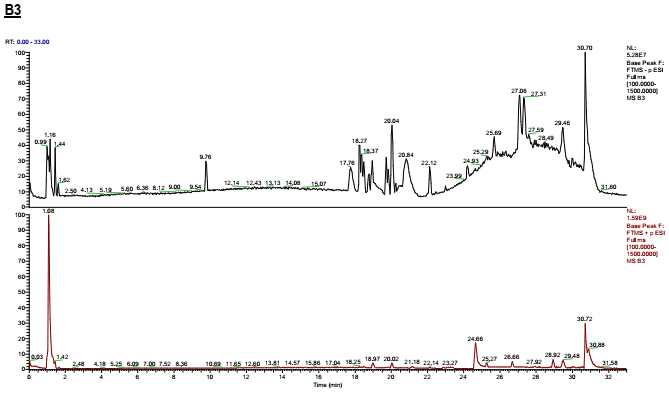 |

**Figure S3. Total Ion Chromatogram (TIC): Beef Patty 1.0% Sacha Inchi leaf extracts**

| 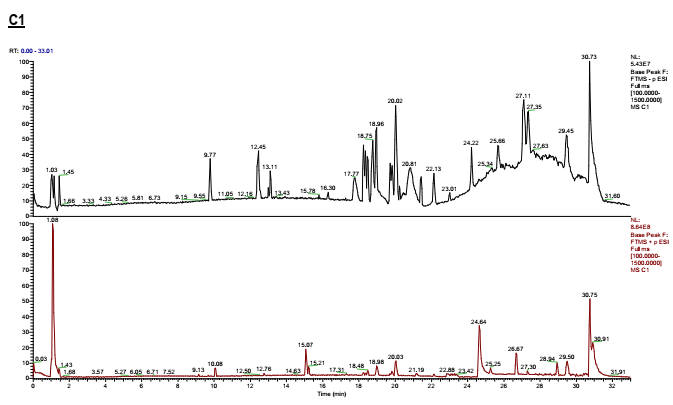 |
| --- |
| 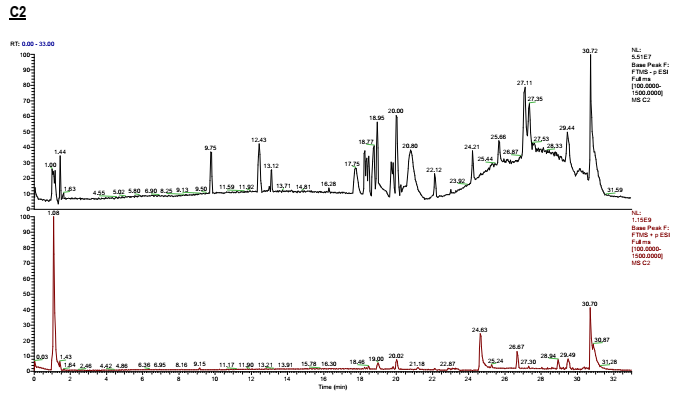 |
| 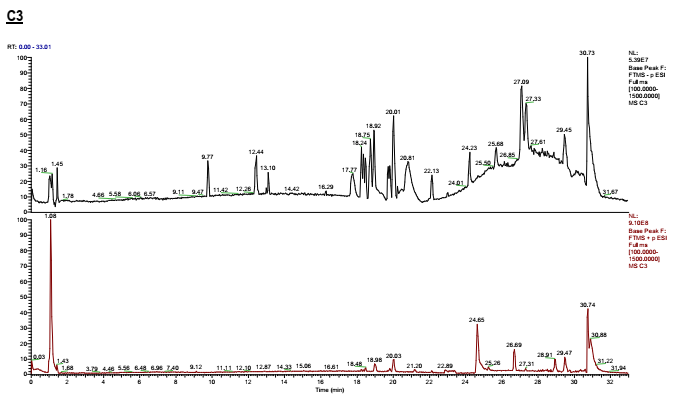 |

**Figure S4. Total Ion Chromatogram (TIC): Beef Patty 1.5% Sacha Inchi leaf extracts**

| 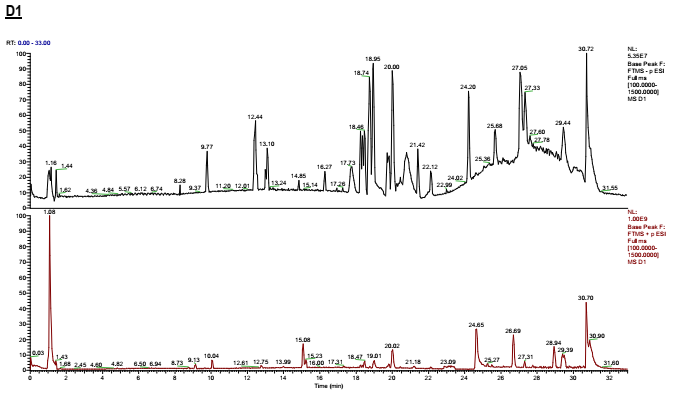 |
| --- |
| 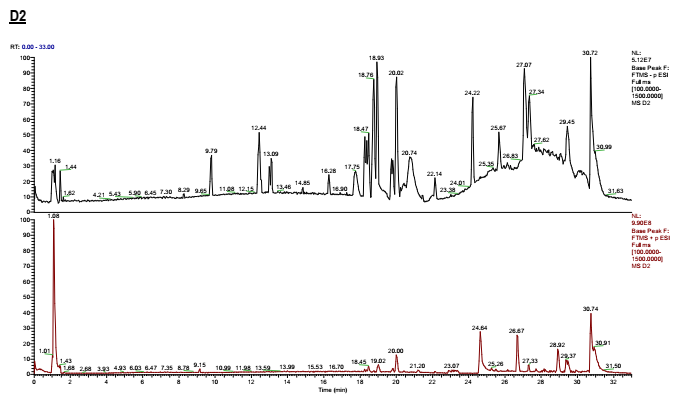 |
| 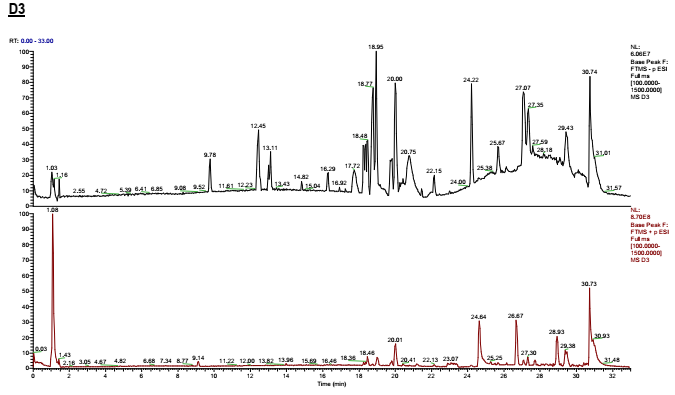 |
